# A comprehensive analysis of the SARS-CoV-2 omicron variant in Tocantins State, Brazil, and tracing the spread of the XBB.1.18.1 lineage

**DOI:** 10.1007/s42770-026-01884-1

**Published:** 2026-03-23

**Authors:** Mateus Silva Santos, Ueric José Borges de Souza, Franciano Dias Pereira Cardoso, Jucimária Dantas Galvão, Fernando Rosado Spilki, Célia Maria de Almeida Soares, Fabrício Souza Campos

**Affiliations:** 1 Bioinformatics and Biotechnology Laboratory, Campus of Gurupi, Federal University of Tocantins, Gurupi, Tocantins 77410-570 Brazil; 2 Tropical Medicine Post-Graduate Program, Federal University of Goiás, Goiânia, Goiás 74690-900 Brazil; 3 Central Public Health Laboratory of the State of Tocantins, Palmas, 77054-970 Brazil; 4 Molecular Microbiology Laboratory, Feevale University, Novo Hamburgo, 93525-075 Brazil; 5 Molecular Biology Laboratory, Institute of Biological Sciences, Federal University of Goiás, Goiânia, Goiás 74690-900 Brazil; 6 Bioinformatics and Biotechnology Laboratory, Department of Microbiology, Immunology, and Parasitology, Institute of Basic Health Sciences, Federal University of Rio Grande do Sul, Porto Alegre, 90050-170 Brazil

**Keywords:** SARS-CoV-2, Genome sequencing, Phylogenetic analysis, Virus transmission, Public health, Genomic surveillance

## Abstract

This study presents a genomic and epidemiological exploration of the omicron variant sublineages in Tocantins, Brazil. A total of 556 positive samples, obtained from individuals testing positive for SARS-CoV-2 between December 21, 2021, and June 30, 2023, underwent genome sequencing. Following RNA extraction, cDNA synthesis was executed, and a multiplexed PCR assay was employed for sequencing using the Oxford Nanopore MinION platform. The dispersal patterns of the XBB.1.18.1 lineage were analyzed as a case study, employing maximum likelihood phylogenetic tree construction and a discrete phylogeographical model. Subsequent analyses unveiled 39 sublineages, with BQ.1.1, BA.5.2.1, and BA.1.14.1 emerging as the most prevalent over the studied period. Notably, the emergence of the BQ.1.1 and BQ.1 lineages in Tocantins was observed in November and December 2022, respectively. The XBB.1.18.1 lineage, dominant from January to June 2023, exhibited noteworthy local and international circulation. Through mapping the migration patterns of this lineage, Tocantins was identified as a pivotal node for virus dispersal, with minimal importation cases. The study estimated the global emergence of the XBB.1.18.1 lineage in mid- to late-October 2022. This investigation contributes to comprehending SARS-CoV-2 dynamics in Tocantins and underscores the significance of transportation routes in virus propagation.

**Supplementary Information:**

The online version contains supplementary material available at 10.1007/s42770-026-01884-1.

## Introduction

The Severe Respiratory Syndrome Coronavirus 2 (SARS-CoV-2), the causative agent of COVID-19, precipitated a global pandemic with unprecedented consequences for the health and economies of numerous countries. By the close of 2023, COVID-19 had resulted in over 770 million confirmed cases and 6.8 million deaths globally [1]. Despite vaccination campaigns being conducted across all continents, certain revelations regarding the infection have posed new challenges for researchers. Adaptive mutations in the viral genome have the potential to modify the pathogenicity of the virus, significantly impacting its ability to evade the immune system, necessitating ongoing updates to vaccines [2, 3].

SARS-CoV-2 has demonstrated a propensity for genetic evolution as it adapts to its new human host, accumulating mutations over time, leading to the emergence of multiple variants, some of which are classified as Variants of Concern (VOCs) due to their impact on public health [4]. VOCs are linked with heightened transmissibility or virulence, increased capacity to evade detection and decreased neutralization by antibodies acquired through natural infection or vaccination, thereby diminishing vaccine effectiveness [5].

Currently, among SARS-CoV-2, the Omicron variant (B.1.1.529) stands out prominently. First detected in the Gauteng province, South Africa, on November 24, 2021, through genomic sequencing and epidemiological analysis [6], it rapidly attracted global attention due to its high transmissibility, making it a major public-health concern [5–7]. Characterized by more than 30 substitutions in the spike (S) protein relative to earlier VOCs, Omicron harbors antigenic changes that increase transmissibility and permit partial escape from infection- or vaccine-elicited antibodies, leading to a higher frequency of breakthrough infections and reinfections and lower effectiveness against infection [7]. Nevertheless, updated vaccines and boosters continue to markedly reduce hospitalizations and deaths, whereas protection against infection is lower and wanes over time; accordingly, periodic boosting with variant-adapted formulations, together with sustained genomic and antigenic surveillance, remains essential to preserve high protection and to inform next-generation vaccines with broader, more durable immunity [8, 9].

The emergence of new Omicron lineages has attracted significant attention and concern worldwide. In addition to the higher mutation rate, it also presents significant changes in its genetic makeup, which may impact the real-world efficacy of available vaccines [10, 11]. Among these variants, the XBB lineage is particularly notable, emerging around July 2022 through recombination events involving two descendants of BA.2, BJ.1, and BM.1.1.1, a progeny of BA.2.75 [12–15]. Its initial spread was primarily in Asia, with Singapore experiencing a sharp rise in cases attributed to this strain. XBB inherited the 5’ part of its genome from BJ.1 and the 3’ end from BA.2.75, with a single breakpoint within Spike’s RBD. This unique breakpoint within the Spike protein confers potent antigenic RBD mutations characteristic of both BJ.1 and BA.2.75, rendering it one of the most potent combinations of antigenic RBD mutations observed among variants to date, resulting in a relatively substantial antigenic divergence from preceding variants [16].

The initial prevalence of the XBB lineages in Brazil was characterized by strains carrying the mutation S: F486P. The predominant XBB*+F486P lineages that emerged and spread within Brazil included XBB.1.5.86, XBB.1.5.102, XBB.1.18.1, and XBB.1.4.2. Since late 2022, XBB* lineages have gained dominance, exhibiting enhanced binding to human ACE2 while maintaining a remarkably strong ability to evade humoral immunity [17–20]. These immunoevasive lineages continuously accumulate S mutations, such as R403K, V445S, L455F, F456L, and K478R, which may result in a more significant change in antigenicity and the evasion of neutralizing antibodies induced by repeated vaccination and previous infection [16, 21]. Notably, certain immune escape mutations, exemplified by F456L, have recently emerged in a convergent manner across multiple independent strains of XBB derivatives, such as EG.5, XBB.1.5.10, FE.1, and FD.1.1, indicating strong selection pressure due to herd immunity [22, 23].

Therefore, genomic surveillance studies play a pivotal role in investigating SARS-CoV-2 and its variants, providing crucial information to understand viral evolution, dissemination, and public health impacts [24]. Leveraging Oxford Nanopore sequencing enables rapid, cost-aware detection of mutations that may alter transmissibility or disease severity and facilitates real time tracking of variant spread, including in resource limited settings [25–27]. In Tocantins, Brazil, the first Omicron case was officially reported on 10 December 2021 [26, 28]. Tocantins is a strategic mobility corridor connecting Brazil’s Southeast, Central-West, North, and Northeast regions via major road and air routes (e.g., the Belém–Brasília highway), creating conditions for repeated viral introductions and onward spread [25–27]. Prior genomic work has already implicated Tocantins in the import of P.1.7 from São Paulo and subsequent exports to Goiás, Mato Grosso, Amapá, and Pará, as well as in the nationwide diffusion of AY.99.2 [26]. These factors highlight the need for sustained genomic and epidemiological monitoring in a state that functions as a mobility corridor and shows heterogeneous sequencing coverage relative to epidemic burden. To characterize the viral landscape in Tocantins, we conducted a genomic and epidemiological investigation focusing on Omicron sublineages. Here, we address an underexplored gap by focusing on the XBB.1.18.1 lineage in Tocantins. We demonstrate the state’s outsized contribution to this lineage and reconstruct introductions and exports to other Brazilian regions and to multiple international destinations, situating these movements within regional and national sublineage turnover and motivating targeted genomic surveillance along mobility corridors.

## Materials and methods

### Sample collection

Samples obtained from both symptomatic and asymptomatic patients in the state of Tocantins underwent testing for SARS-CoV-2 infection status via real-time reverse-transcription polymerase chain reaction (RT-qPCR). This assay was conducted on nasal swab samples using a commercial kit (SARS-CoV-2-E/RP, Biomanguinhos), provided by the Brazilian Ministry of Health, and utilized as standard practice across all public health laboratories in Brazil. Samples collected from patients testing positive for SARS-CoV-2 between December 21, 2021, and June 30, 2023, were then subjected to genome sequencing. A total of 556 positive samples, with a quantification cycle (*Cq*) of less than 29 for at least one primer, were selected and submitted to SARS-CoV-2 genome sequencing. The study was approved by the Ethics Committee (33202820.7.1001.5348).

### RNA extraction and sequencing

Sample processing was conducted at the Central Public Health Laboratory of the State of Tocantins (LACEN-TO) using a magnetic beads-based protocol with an Extracta^®^ Viral RNA MVXA-P096 FAST kit (Loccus^tm^, Brazil) and an automated extractor (Extracta^®^ 96, Loccus^tm^, Brazil) following the manufacturer’s instructions. cDNA synthesis was performed using Luna Script RT SuperMix (5×) (New England Biolabs, Ipswich, MA, USA). The synthesized cDNAs served as templates for a multiplexed PCR assay employing the ‘Midnight’ SARS-CoV2 genome sequencing protocol (10.17504/protocols.io.14egn2q2yg5d/v1, accessed on August 12, 2023), with 1200 bp amplicon primer sets tailored for long-read sequencing. This approach generated tiled overlapping amplicons covering almost the complete SARS-CoV-2 genome.

The amplicons from both primer sets were combined and purified with a 1x volume of Ampure^®^ XP beads (Beckman Coulter^tm^, Brea, CA, USA). Library preparation for MinION^®^ sequencing was performed using the rapid barcoding kit (SQK-RBK110.96). Rapid barcodes were added to the 96-well Barcode Attachment Plate by mixing 2.5 µl nuclease-free water, 5 µl pooled PCR products (from pools A and B), and 2.5 µl barcodes from the Rapid Barcode Plate. All samples were pooled and cleaned up using magnetic SPRI beads. Rapid adapters were added to 600–800 ng of the library, mixed, and incubated for 5 min. Subsequently, the sequencing buffer, loading beads, and DNA library were mixed and loaded onto Oxford Nanopore MinION^®^ SpotON Flow Cells FLO-MIN106D, R9.4.1 (Oxford Nanopore Technologies^tm^) and subjected to sequencing using the MinION Mk1B device.

High-precision base calling was performed using Guppy v6.5.7 (Oxford Nanopore Technologies). The assembly of high-precision base called fastq files was conducted using the bioinformatics protocol of the novel coronavirus nCoV-2019 (https://artic.network/ncov-2019/ncov2019-bioinformatics-sop.html, accessed on August 12, 2023) with Minimap2 [29] and Medaka (https://github.com/nanoporetech/medaka, accessed on August 12, 2023) for consensus sequence generation. The resulting genomes underwent quality control and were classified into viral genome clades using the Nextclade version 2.14.1 [30]. Lineages were assigned to each genome using Pangolin v4.3.1 [31]. All Tocantins genomes generated in this study have been submitted to GISAID.

### Epidemiological analysis

The course of the COVID-19 pandemic in Tocantins from November 1, 2021, to June 30, 2023, was evaluated by compiling data on the number of cases and deaths attributed to the disease. This analysis utilized publicly accessible information from the Secretary of Health of Tocantins (http://integra.saude.to.gov.br/covid19/InformacoesEpidemiologicas/, accessed on April 1, 2024). Furthermore, the prevalence of each viral lineage during the specified period in Tocantins was investigated using data sourced from both GISAID and newly sequenced genomes. Graphical representations were generated using the ggplot2 package [32] within the R software (version 4.3.2).

### Phylogenetic analysis

To assess the spread of SARS-CoV-2 across Tocantins, a detailed analysis of dispersal patterns within the XBB.1.18.1 lineage was conducted. Sequences of XBB.1.18.1 available up to September 30, 2023, were downloaded from the GISAID database (accessed on February 16, 2024). We retained human clinical genomes assigned to XBB.1.18.1 (Pango) that met basic quality thresholds (genome length ≥ 29,000 nt; ambiguous bases ≤ 5%; no obvious sequencing artifacts upon manual inspection) and that had collection date available at least to year–month (using exact dates when available). Multiple sequence alignment was conducted using MAFFT v7.505 [33] with default settings, followed by manual inspection using AliView v1.28 [34]. The maximum likelihood (ML) phylogenetic tree was built using IQ-TREE2 v2.2.2.6 [35]. The IQ-TREE2 v2.2.2.6 analysis was executed under the generalized time-reversible (GTR) model of nucleotide substitution with empirical base frequencies (+ F) and the invariable site plus FreeRate model (I + R4), determined by the ModelFinder v1.4.2 [36] and an SH-aLRT branch test (− alrt 1000). Subsequently, the ML tree was examined in TempEst v1.5.3 [37] to evaluate the presence of a temporal or molecular clock signal. Outlier sequences displaying strong deviations were excluded, resulting in a final dataset of 1,073 sequences (Supplementary Fig. 1). A time-scaled phylogenetic tree was inferred using TreeTime v0.11.0 [38]. The annotated tree topology was then used to estimate the number of viral transmission events in Tocantins between Brazilian regions and the rest of the world, employing the TreeTime v0.11.0 “*mugration*” model. Visualizations of the time-resolved phylogenetic tree were generated in R (version v4.1.2) using the ggtree package [39].

### Bayesian analysis

To infer the substitution rate of the XBB.1.18.1 lineage, we utilized the new and faster tree likelihood algorithm available in BEAST v1.10.5 (pre-release Thorney v0.1.1) package [40], coupled with the skygrid coalescent prior [41]. All remaining priors and operators were set as defined in default. We conducted six independent Markov Chain Monte Carlo (MCMC) runs, with each chain comprising 100,000,000 steps and sampling occurring every 10,000th step. We removed a burn-in of 10% of the posterior trees and summarized the results using TreeAnnotator v1.10.5 [40]. Convergence and mixing were assessed in Tracer v1.7.2, and we required effective sample size (ESS) > 200 for all key parameters (posterior, likelihood, clock rate, substitution model and tree prior parameters).

Additionally, we extracted sequences from three subclades that prominently clustered numerous Tocantins sequences from the phylogenetic tree. Multiple sequence alignment was conducted using MAFFT v7.505 with default settings, followed by manual inspection using AliView v1.28. The temporal signal assessment was performed with TempEst v1.5.3 (Supplementary Figs. 2–4). Spatial and temporal patterns of diffusion were estimated through a Bayesian Markov Chain Monte Carlo (MCMC) approach implemented in BEAST v1.10.4 [40] with BEAGLE v3.1.2 [42] to enhance computational efficiency. The GTR + G + I model was employed to model nucleotide evolution under a strict clock model with a Continuous Time Markov Chain (CTMC) and the exponential coalescent model [43]. For each dataset, three independent MCMC chains were executed, each running for 50 million states, with sampling at every 5,000 generations. The results of these independent runs were merged using LogCombiner v1.10.4 [40], and the convergence of the MCMC chain was assessed using Tracer v1.7.2 [44]. Maximum clade trees were summarized from the MCMC samples using TreeAnnotator V1.10.4, discarding the initial 10% as burn-in [40]. We required ESS > 200 after merging and the MCMC phylogenetic trees were visualized using the ggtree R package [39].

A discrete phylogeographical model [45] was employed to reconstruct the spatial diffusion of the virus across the compiled dataset’s sampling locations. Phylogeographic analyses involved applying an asymmetric model of location-transitioning, and the estimation of location diffusion rates was carried out using the Bayesian stochastic search variable selection (BSSVS) model with a discretization scheme defined at the level of Brazilian states and international countries. MCMC was run sufficiently long to ensure stationarity and achieve an effective sample size (ESS) exceeding 200.

## Results

Of the total of 556 samples, 62.4% (347) were female and 37.6% (209) were male, with 6.6% (37) falling within the age group up to 19 years, 76.1% (423) among individuals aged between 20 and 59 years, and 17.3% (96) aged 60 years and over. The selected samples exhibited Ct (cycle threshold) values ranging from 11.6 to 29.1, with a mean of 21.9.

**Fig. 1:**
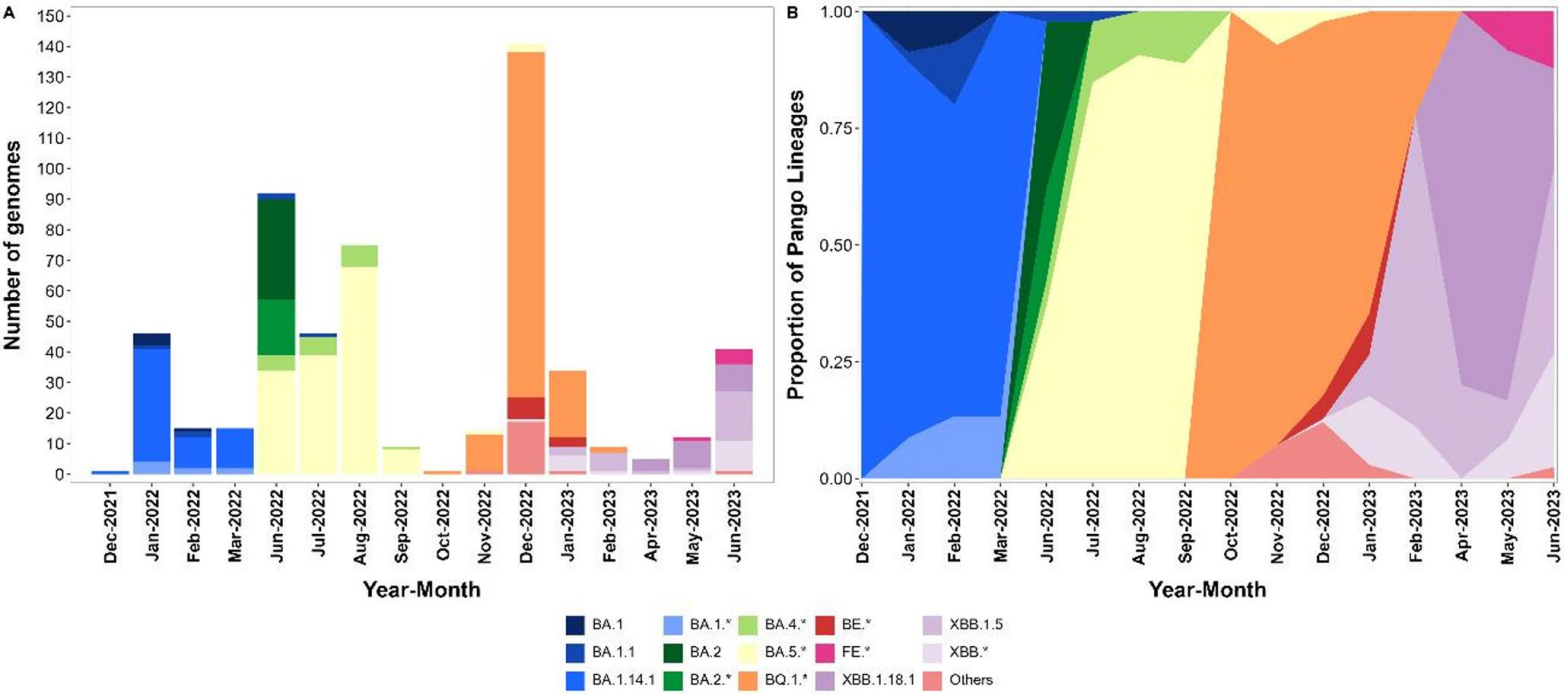
Omicron subvariant dynamics in Tocantins between December 2021 to June 2023 based on our collected sequences (monthly counts on the left; relative frequencies on the right). Labels with “*” group a parent lineage with all sublineages; “Others” pools low-frequency sublineages

In total, 39 Pango lineages were observed in the 556 sequenced genomes. The most frequently detected lineage was BQ.1.1 (100 sequences, 18%), followed by BA.5.2.1 (79 sequences or 14.2%), BA.1.14.1 (61 sequences or 11.0%), BQ.1 (47 sequences or 8.5%), BA.5.1 (38 sequences or 6.8%), BA.2 (33 sequences or 5.9%), XBB.1.5 (27 sequences or 4.9%), BA.5 (26 sequences or 4.7%), XBB.1.18.1 (22 sequences or 4.0%) (Fig. 1).

**Fig. 2:**
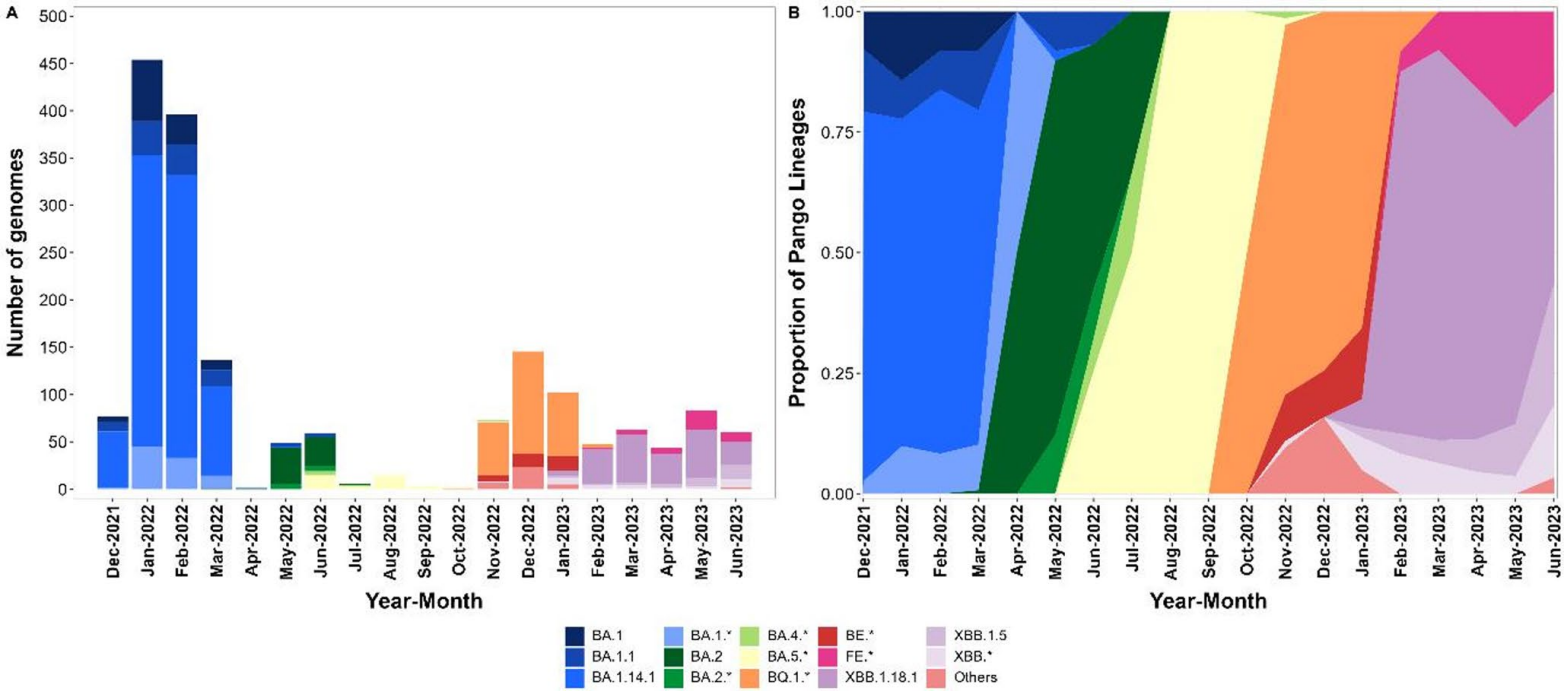
Omicron subvariant dynamics in Tocantins between December 2021 to June 2023 using sequences deposited in GISAID (monthly counts on the left; relative frequencies on the right). “*” indicates aggregation by parent lineage; “Others” pools low-frequency sublineages

As depicted in Fig. 1, based on our data collection, we highlight that between December 2021 and March 2022, the sequenced genomes indicated the presence of BA.1 lineages and BA.14.1 as the main circulating lineages during this period. However, by June 2022, BA.2 lineages emerged as predominant, followed by BA.5.1, BA.5.2.1, BA.5, and BA.4 lineages. These findings align with the strains isolated in Tocantins available in the GISAID database during the same period (Fig. 2), suggesting the arrival of these lineages in the state as of March 2022.

We noted that November 2022, particularly December 2022, marked the introduction of the BQ.1.1 and BQ.1 lineages in the state, as evidenced by repeated detections across consecutive months and spread beyond initial sampling sites, consistent with community transmission (Fig. 1). During January–June 2023, our surveillance captured additional introductions, notably XBB.1.18.1 and XBB.1.5, accompanied by an increase in relative abundance that suggests ongoing replacement of circulating Omicron sublineages, potentially affecting COVID-19 epidemiology in Tocantins.

**Fig. 3:**
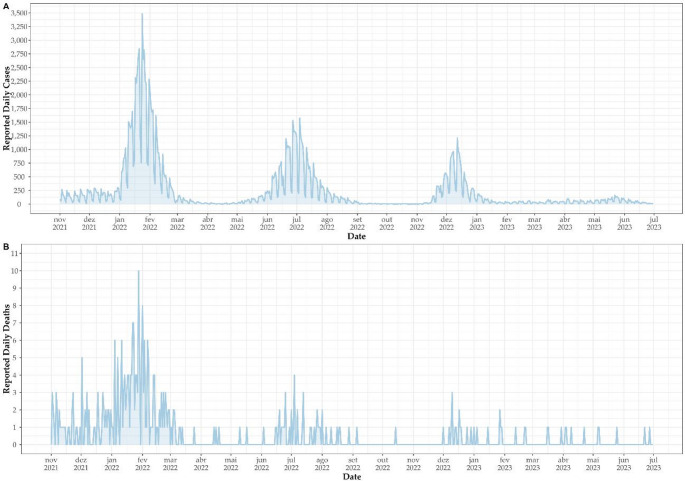
Evolution of the COVID-19 pandemic in Tocantins between November 1, 2021, to June 30, 2023. Panel (**A**) represents the absolute number of cases in this period, while (**B**) is the absolute number of deaths

Analysis of epidemiological data reveals a notable trend in COVID-19 cases in Tocantins from November to December 2021 (Fig. 3), with a marked decline in cases, reaching the lowest levels observed since the onset of the pandemic in the state (957 COVID-19 cases in the third week of December 2021). Notably, during this period, the Delta variant predominated. However, as the year transitioned, case numbers began to rise once more, peaking at over 14,920 cases reported during the fourth week of January 2022, marking the highest count recorded in Tocantins up to the time of this study (Fig. 3A). This resurgence was particularly influenced by the emergence and spread of the Omicron variant in the state, alongside BA.1 lineages and sublineages. In June 2022, a resurgence in case numbers was observed, primarily driven by BA.2, BA.4, and BA.5 lineages (Fig. 3A). Additionally, between December 2022 and January 2023, case numbers surged once again, influenced by the emergence of XBB lineages (Fig. 3A). Correspondingly, there was a notable increase in deaths, reaching 35 during the fourth week of January, the highest recorded during the study period (Fig. 3B), albeit lower than the peak observed in the first half of 2021 when the Gamma variant was spreading across Brazil (not depicted in Fig. 3).

To enhance our understanding of the XBB.1.18.1 lineage and its implications for virus dissemination in Tocantins, we undertook comprehensive analyses. Reviewing GISAID at our data freeze (accessed 16 February 2024; inclusion limited to genomes with collection dates ≤ 30 September 2023), we found that Tocantins accounted for 179 of 1,401 global XBB.1.18.1 genomes (12.8%), indicating a disproportionately high contribution from the state. These findings, combined with our investigations, suggest that the state likely played a significant role in the dissemination of the XBB.1.18.1 lineage at both national and international scales.

### Phylogenetic analysis

**Fig. 4:**
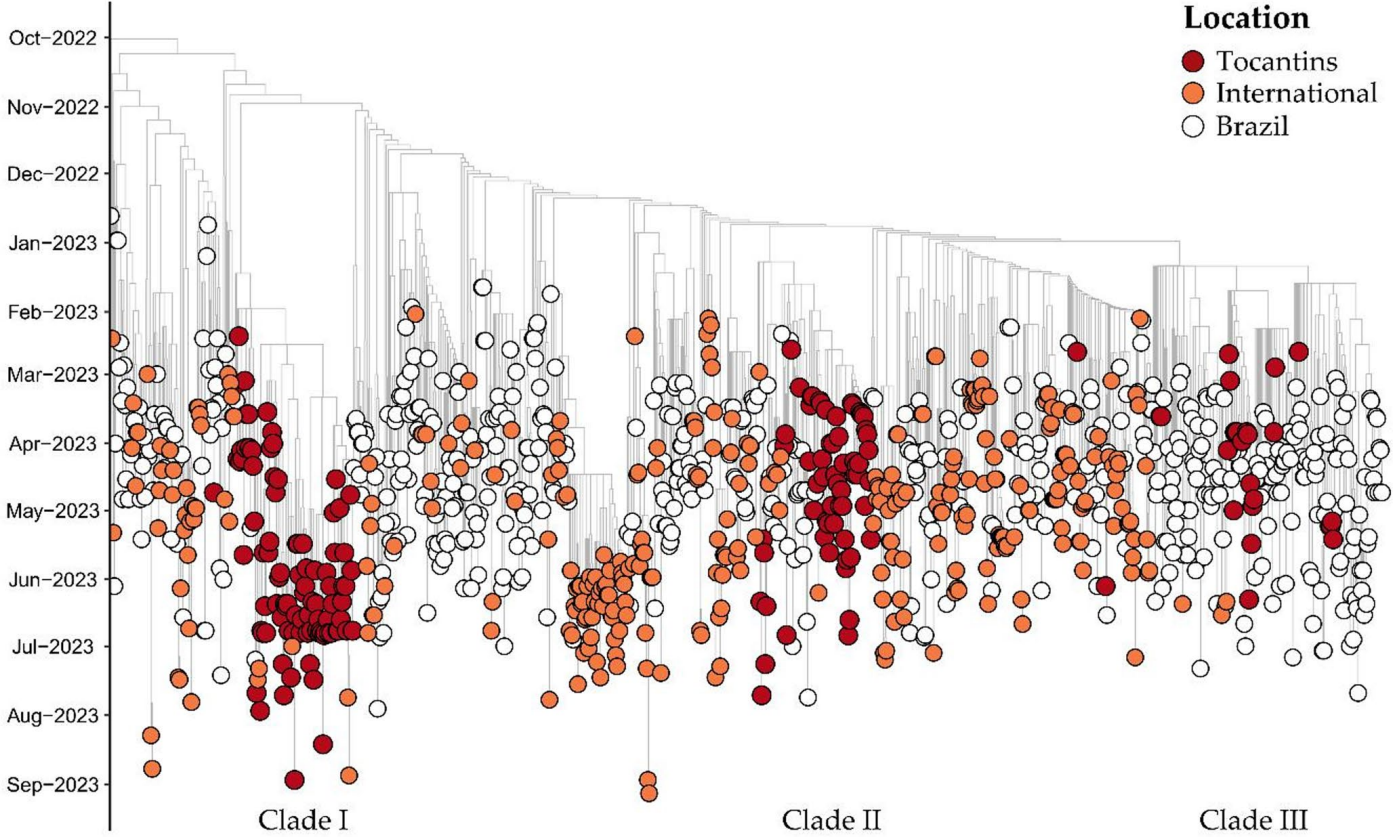
Time-Resolved Maximum-Likelihood Tree of 1073 SARS-CoV-2 Genomes of the XBB.1.18.1 Lineage. Tips represent sequences from both Brazil and international countries, with genomes from Tocantins highlighted in red

Phylogenetic analysis was performed on 1073 genomes of XBB.1.18.1, adhering to the classifications of the PANGOLIN lineage and GISAID clades. The time-resolved maximum likelihood phylogenetic tree depicted in Fig. 4 illustrates that sequences from the Tocantins state formed three distinct subclades, predominantly originating from Brazil with some contributions from international sources (Fig. 4). The phylogenetic tree was rooted in a genomic sequence obtained from São Paulo state in November 2022 (EPI_ISL_16372004). The earliest genome of this lineage in Tocantins was identified in January 2023, clustering within Clade I (Fig. 4).

It is notable that out of the total genomes utilized for phylogenetic reconstruction, 179 (16.7%) were sourced from Tocantins, representing the largest number of genomes available for this lineage. This underscores a significant circulation of this lineage from January 2023 to June 2023, as illustrated in Figs. 1 and 2 above. Tocantins was followed by genomes obtained from São Paulo in the Southeast of Brazil (*N* = 106; 9.9%), the United States (*N* = 71; 6.6%), Mato Grosso do Sul in the Central-West of Brazil (*N* = 69; 6.4%), Pará in the North of Brazil (*N* = 59; 5.5%), Goiás in the Central-West of Brazil (*N* = 47; 4.4%), and the United Kingdom (*N* = 34; 3.2%).

**Fig. 5:**
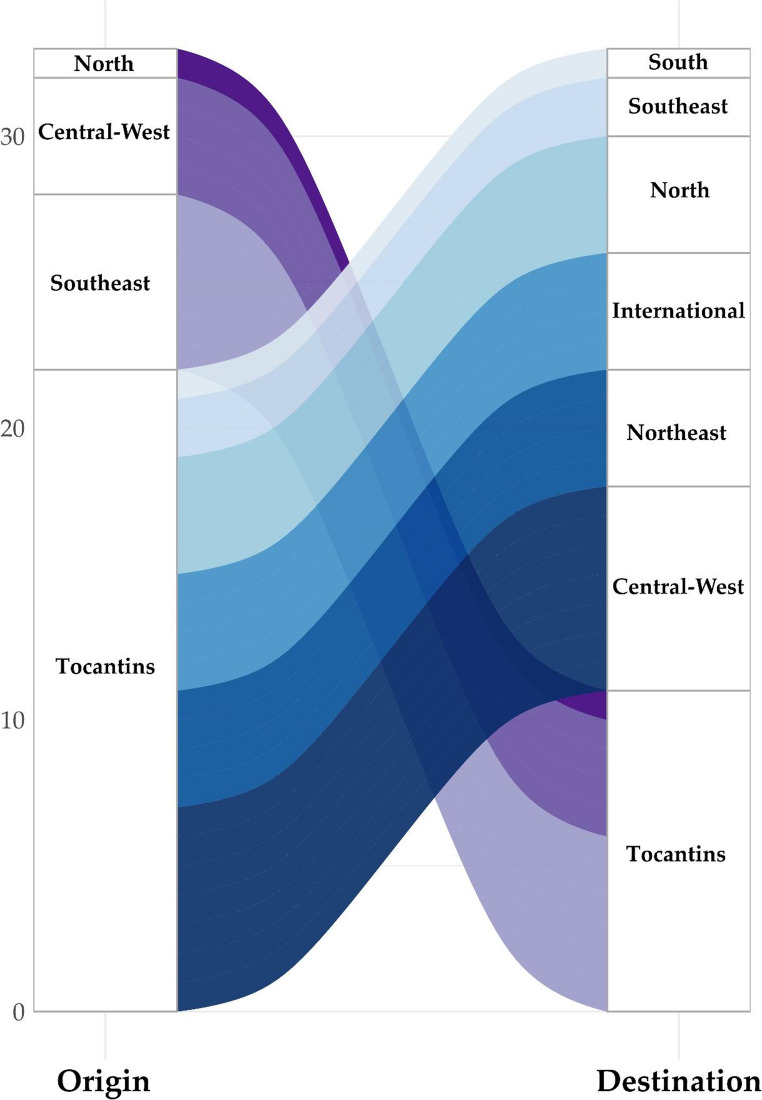
Estimated Dispersal of the XBB.1.18.1 Lineage in Tocantins State through ‘*mugration*’ model. Transmission routes are depicted, with the left bar indicating the location of origin for dispersal and the right bar indicating their destinations

Through the analysis of connections between “origin” and “destination” regions using the ‘*mugration*’ model, we observed that the Tocantins state played a central role in the dispersal to other regions, including international transmission, with a minimal number of importation cases (Fig. 5). Tocantins received importation events of the XBB.1.18.1 lineage from the Southeast region from December 2022 to May 2023 (*N* = 6), from the Central-West region from December 2022 to March 2023 (*N* = 4), and only one event was observed from the North region in January (Fig. 5 and Supplementary Fig. 5). The Central West region received the highest number (*N* = 7) of importing events from Tocantins between December 2022 to May 2023. The Northeast and North regions, as well as countries outside Brazil, each received four introductions from Tocantins (Fig. 5).

We estimated that the XBB.1.18.1 lineage emerged globally as early as mid-to late-October 2022 (95% HPD: September 20, 2022, to November 06, 2022). By mid-November 2022, this lineage was circulating in Brazil and various parts of the world. Our analysis resulted in a median evolutionary rate of 5.01 × 10^− 4^ (95% highest posterior density interval (HDP): 4.68 × 10^− 4^ to 5.30 × 10^− 4^ substitutions per site per year).

**Fig. 6:**
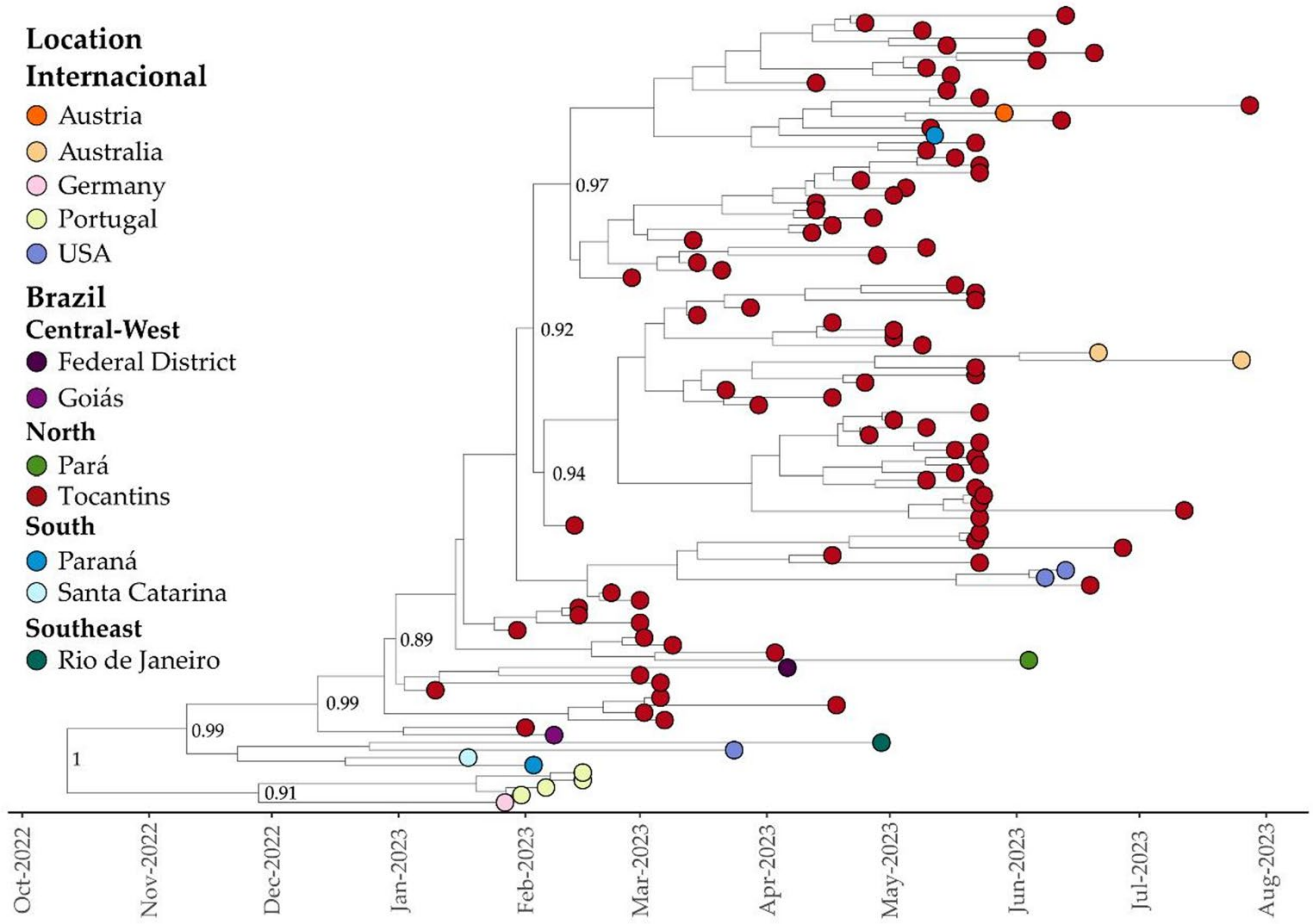
Bayesian Time-Scaled Phylogenetic Tree of 106 XBB.1.18.1 SARS-CoV-2 Samples Across Global Regions. Sampling locations are color-coded as per the legend on the left side of the tree. The genomes from Tocantins, highlighted in this study, are distinguished in red

Within the XBB.1.18.1 lineage, we identified three subclades with robust branch support, encompassing a significant number of sequences primarily from Tocantins. Regarding Clade I, our analysis yielded a median evolutionary rate of 6.34 × 10^− 4^ (95% HDP): 5.03 × 10^− 4^ to 7.81 × 10^− 4^ substitutions per site per year), and the time to the most recent common ancestor (TMRCA) was estimated as October 9, 2022 (95% HPD: August 22, 2022, to November 23, 2022) (Fig. 6). Using the BSSVS procedure, we identified well-supported rates of posterior diffusion originating from Tocantins to countries beyond Brazil, notably the USA (Bayes Factor (BF): 106.19; Posterior Probability (PP): 0.91), Austria (BF: 57.50; PP: 0.85), and Australia (BF: 32.17; PP: 0.76). Additionally, our analysis revealed diffusion rates from the Tocantins to various regions within Brazil, including the South region (Paraná – BF: 55.77; PP: 0.84; and Santa Catarina – BF: 12.86; PP: 0.56), the North region (Pará – BF: 17.33; PP: 0.58), and the Central West region (Federal District – BF: 19.80; PP: 0.66; and Goiás – BF: 16.51; PP: 0.62).

**Fig. 7:**
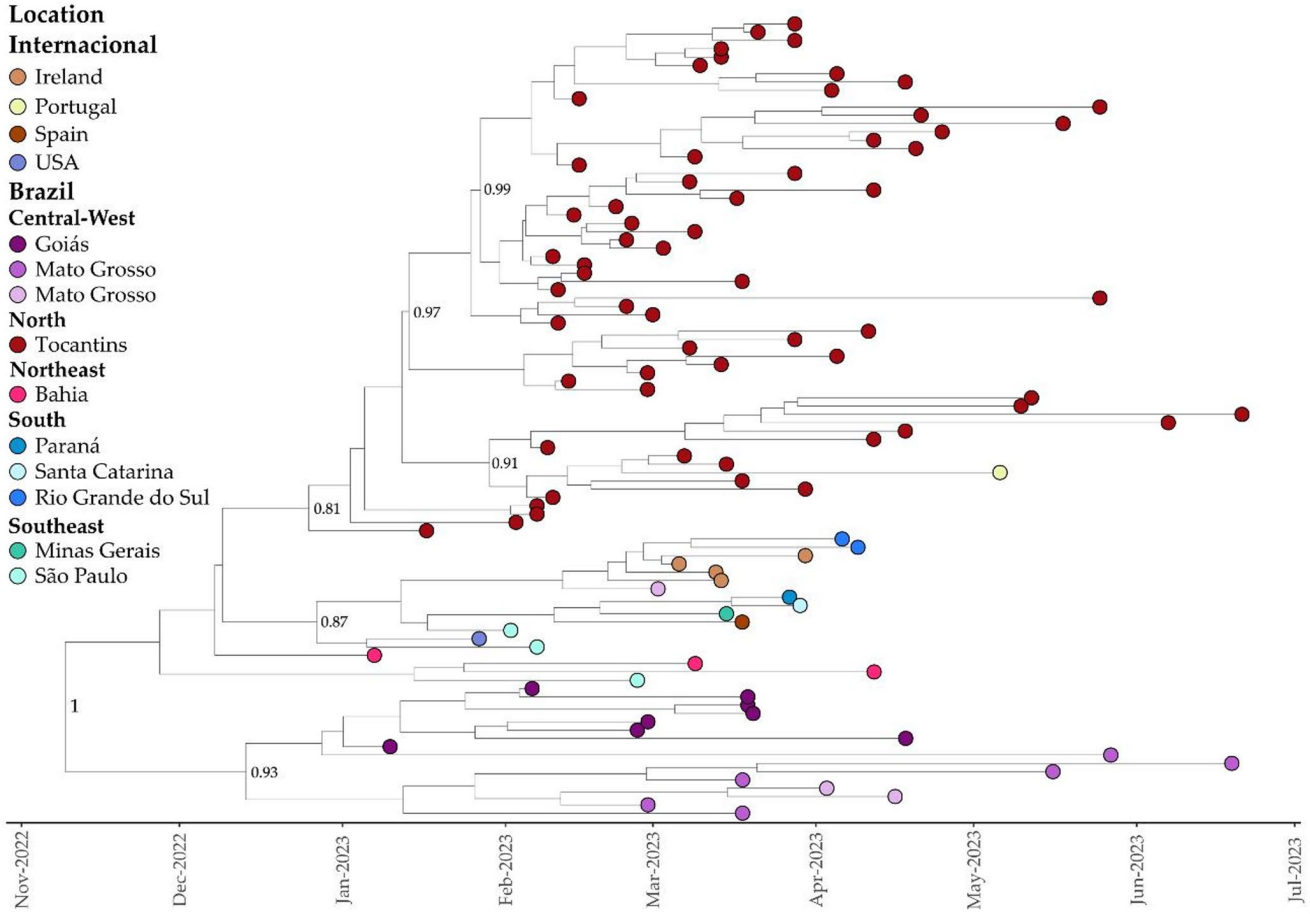
Bayesian Time-Scaled Phylogenetic Tree of 96 XBB.1.18.1 SARS-CoV-2 Samples Across Global Regions. Sampling locations are color-coded as per the legend on the left side of the tree. The genomes from Tocantins, highlighted in this study, are distinguished in red

For Clade II, our analysis yielded a median evolutionary rate of 6.17 × 10 − 4 (95% highest posterior density interval (HDP): 4.68 × 10 − 4 to 7.80 × 10 − 4 substitutions per site per year), with the TMRCA estimated as November 9, 2022 (95% HPD: October 02, 2022, to December 13, 2022) (Fig. 7). Employing the BSSVS procedure, we identified well-supported rates of posterior diffusion originating from Tocantins only to Portugal (BF: 148.28; PP: 0.92). Additionally, we observed significant viral introductions from Bahia to Tocantins (BF: 16.28; PP: 0.57), as well as to São Paulo (BF: 16.09; PP: 0.57), Goiás (BF: 9.49; PP: 0.44), and Mato Grosso (BF: 9.05; PP: 0.42). Beyond the focus of this study, specifically Tocantins, the tree reconstruction also showed important migrations from Mato Grosso to Mato Grosso do Sul (BF: 171.18; PP: 0.93), from Ireland to Rio Grande do Sul (BF: 74.41; PP: 0.86), and from São Paulo to the United States (BF: 55.25; PP: 0.81).

**Fig. 8:**
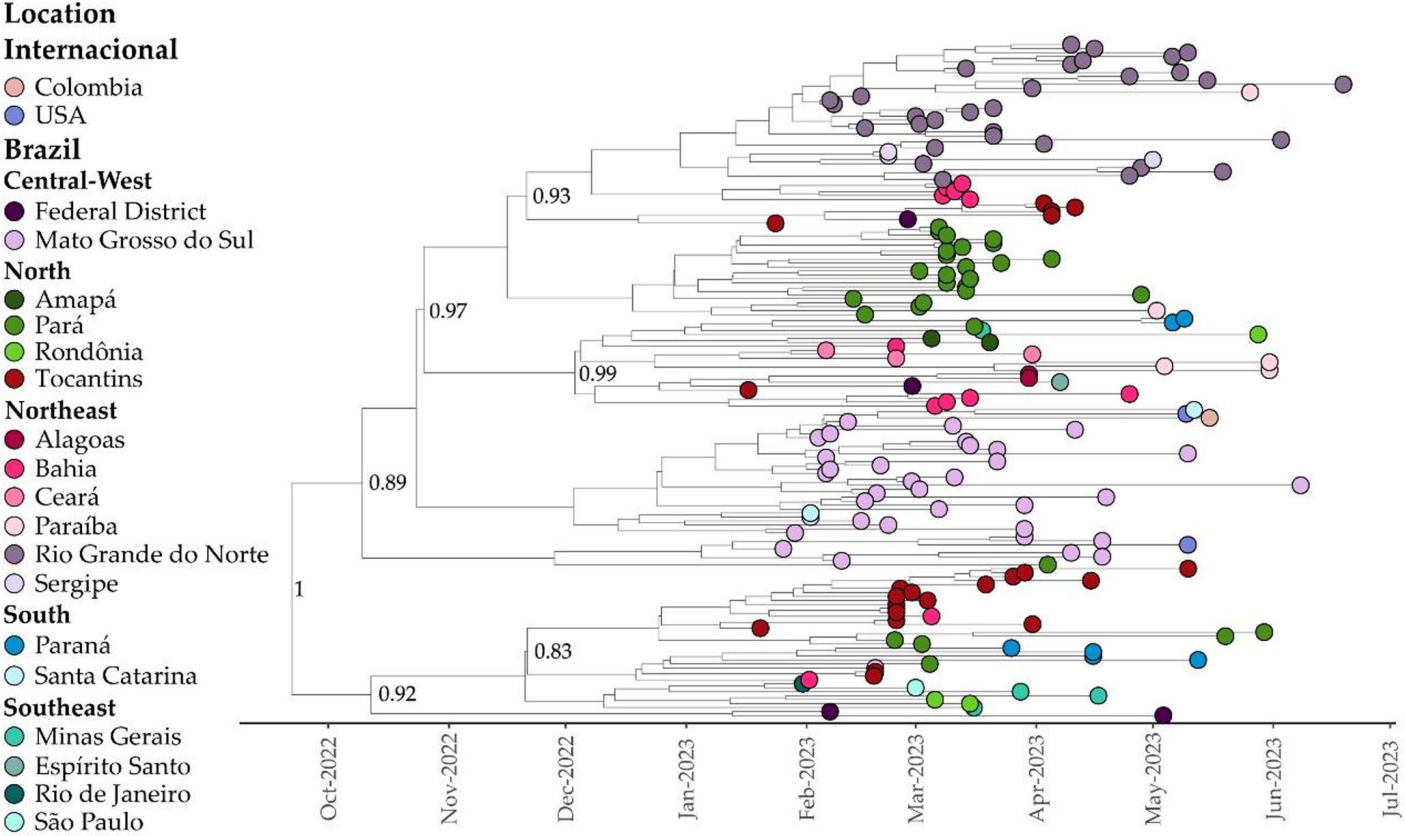
Bayesian Time-Scaled Phylogenetic Tree of 132 XBB.1.18.1 SARS-CoV-2 Samples Across Global Regions. Sampling locations are color-coded as per the legend on the left side of the tree. The genomes from Tocantins, highlighted in this study, are distinguished in red

For the Clade III, the TMRCA was estimated to be September 18, 2022 (95% HPD: July 31, 2022, to November 1, 2022), with a median evolutionary rate of 5.44 × 10 − 4 (95% HPD: 4.32 × 10 − 4 to 6.58 × 10 − 4) (Fig. 8). The BSSVS procedures showed well-supported rates of posterior diffusion originating from the Tocantins to the North region (Pará – BF: 109.37; PP: 0.86), to the Northeast region (Bahia – BF: 75.91; PP: 0.81) to the Central-West region (Federal District – BF: 183.52; PP: 0.91 and Mato Grosso do Sul – BF: 54.57; PP: 0.75), to the South region (Paraná – BF: 22.20; PP: 0.55) and the Southeast region (Minas Gerais – BF: 23.01; PP: 0.56). Finally, the tree reconstruction also showed important migrations from Mato Grosso do Sul to the United States (BF: 107.22; PP: 0.85) and Santa Catarina (BF: 70.96; PP: 0.79), from Pará to Paraíba (BF: 75.96; PP: 0.81), and from Rio de Janeiro to the Sergipe (BF: 72.14; PP: 0.79). Furthermore, across all three clades, it was observed that sequences from Tocantins predominantly grouped in the same clade, indicating a potentially high level of local transmission between cities within the state.

## Discussion

In this study, we present findings from molecular epidemiological research of SARS-CoV-2 conducted in Tocantins State, Brazil, spanning from December 2021 to June 2023. During this period, successive Omicron sublineages shaped local dynamics, including BA.1 and BA.2 in early 2022, BA.5 and BA.4 through mid-2022, BQ.1 and BQ.1.1 in late 2022, and XBB sublineages, notably XBB.1.5 and XBB.1.18.1, in early 2023. This trajectory is consistent with Brazil as a whole, where 626 distinct lineages were recorded between July 2022 and July 2024, and where BA.5 and BA.4 dominated mid-2022, BQ.1.1 rose in late 2022, and XBB, for example XBB.1.5 and XBB.1.18.1, became prominent in 2023, before GK.1.1 and then JN.1 prevailed from 2023 to 2024 [24]. At the regional scale, profiles in the North and Central West broadly mirrored this sequence, with BA.5 and BA.4 peaks in mid-2022, expansion of BQ.1 and BQ.1.1 in late 2022, and a transition to XBB in early 2023, with only minor timing differences plausibly related to sampling intensity and mobility corridors linking Tocantins to Goiás, Mato Grosso, Pará, and Amapá [24]. Within this context, Tocantins showed an early and relatively high representation of XBB.1.18.1 among XBB sequences, aligning with the national shift to XBB while underscoring the state’s role as a corridor for introductions and onward spread.

The Omicron variant was first identified in Botswana [46]. Subsequently, a few months after the confirmation of the first case, its sublineages were observed by various researchers and acknowledged by the World Health Organization (WHO). On February 22, 2022, the WHO reported two sublineages: BA.1 (B.1.1.529.1) and BA.2 (B.1.1.529.2) [47]. Later, in September 2022, the XBB lineage was identified, originating from recombination events involving two BA.2-derived lineages (BJ.1 and BM.1.1.1), and progressively replaced most of the earlier Omicron strains [20, 48]. XBB variants subsequently replaced previously circulating Omicron variants by early 2023. By October 2023, the major lineages of XBB accounted for approximately 80% of reported viral sequences [49].

The first genome of the lineage XBB.1.18.1 in Tocantins was identified in January 2023. We provide a comprehensive analysis of its diffusion within the state, integrating temporal trends with phylogenetic and phylogeographic evidence. Although this variant circulated for approximately 10 months in Brazil, our results suggest that Tocantins likely played a key role in its spread. Despite acknowledging the influence of international sources in shaping SARS-CoV-2 diversity, our findings reveal that the XBB.1.18.1 lineage exhibited its highest dispersal rates within the country. Notably, the Central-West region of Brazil received the highest number of imports from Tocantins, followed by the Northeast and North, as well as countries outside Brazil. This observed pattern can be elucidated by several key factors. The state of Tocantins has an important trade route with the Central-West of Brazil, and the federal highway trans-Brazilian (BR-153) that crosses the region promotes a large flow of people. This heightened human mobility intensifies the circulation of the virus, increasing the probability of its spread. Beyond highways, regional air links and river transport within the Araguaia–Tocantins basin also contribute to connectivity and potential seeding events, patterns documented for Brazil’s integrated land–air network and for riverine travel in the Amazon. The role of multimodal mobility in COVID-19 dissemination has been shown in Brazil, including spread along highways and hubs, air transport networks, and passenger riverboats serving remote communities [26, 50–54].

When assessing the introduction and spread of the variant in Tocantins, it is important to also take into account international routes. Previous studies have shown a particular vulnerability in Brazil to potential unregulated international migration from neighboring countries, principally Venezuela, as well as Colombia, Peru, Bolivia, Paraguay, and Argentina, and the consequent importation of vaccine-preventable diseases [55–60]. Notably, during our study window these countries reported the same Omicron sublineage turnover seen in Brazil, with BQ.1 in late 2022 followed by XBB and XBB.1.5 from early 2023 [58–60]. These patterns support plausible indirect seeding via national hubs linked to international gateways. Historical precedents include measles reintroductions into northern Brazil linked to cross-border migration from Venezuela in 2018 and 2019 [61].

The issue of internal transportation routes in Brazil, which are used for the movement of goods and people and subsequently facilitate the spread of viruses, warrants further investigation. A range of studies have demonstrated this phenomenon with diseases such as dengue [27], CHIKV [25], and even SARS-CoV-2 [26]. In all these cases, major cities, particularly São Paulo and Rio de Janeiro, serve as hubs for receiving viruses via international routes and disseminating them throughout Brazil. Given our findings of repeated introductions and onward transmission of emerging lineages via Tocantins—a state intersected by major logistic routes—we recommend reinforcing surveillance at strategic points along transport corridors such as the BR-153 highway. This could include routine genomic sequencing at sentinel sites (e.g., regional hospitals, border municipalities, or rest stops) to enable early detection of novel variants and monitor their dissemination. Additionally, these data can inform targeted booster vaccination efforts in municipalities with high mobility or low vaccine coverage, allowing for rapid immunization campaigns in areas at greater risk of viral introduction and spread. Such strategies may enhance preparedness and early response capacity, particularly in states like Tocantins that function as epidemiological bridges between Brazil’s northern and central-western regions.

With 77.86% of the population in Brazil having received two doses of the COVID 19 vaccine [62], the country must remain vigilant to avoid importing variants from settings with lower vaccination rates. This calls for enhanced measures at airports and major bus terminals focused on entry screening, symptom monitoring, and genomic surveillance, rather than vaccination delivery. Vaccination should continue to be delivered through primary health care posts and state and municipal campaigns, with microplanning guided by routine data. In Tocantins, state surveillance data (Integra Saúde Tocantins) indicate that coverage rose quickly in 2021 for the primary series, while booster uptake was lower and uneven from 2022 to 2024, mirroring national patterns as shown in Supplementary Fig. 6. These patterns support targeted booster campaigns in municipalities with lower coverage and surge vaccination in specific weeks or areas when routine data indicate declining booster uptake (Supplementary Fig. 6).

The ancestral reconstruction and phylogeographic analysis utilized in this study enabled us to identify well supported rates of posterior diffusion originating from Tocantins to countries beyond Brazil, particularly the USA. While limited literature exists specifically on the XBB.1.18.1 lineage, other studies have provided awareness of the circulation of XBB lineages in the USA. Ma et al. [63] highlighted in their study that as of mid-May 2023, the commonly circulating Omicron lineages were XBB.1.5 (61.5%; 95% CI = 56.4%–66.4%), XBB.1.9.1 (10.0%; 95% CI = 6.8%–14.1%) and XBB.1.16 (9.4%; 95% CI = 6.9%–12.5%), with approximately a combined prevalence of 19% of other circulating lineages, including XBB (5.3%), XBB.1.9.2 (4.5%), XBB.2.3 (3.2%), XBB.1.16.1 (2.4%), and XBB.1.5.1 (1.9%). Ramaiah et al. [64], using maximum likelihood phylogenetic and genetic sequence comparison approaches, identified the transmissibility of specific strains of XBB.1.5 circulating in the populations of Southeastern Wisconsin, USA. Consistent with our findings, CDC genomic surveillance reported BQ.1 and BQ.1.1 predominating in late 2022 and XBB.1.5 becoming predominant in the USA by the end of January 2023, that is, earlier or broadly contemporaneous with Brazil, which supports that our inferred diffusion concerns particular XBB clades such as XBB.1.18.1 rather than the origin of the broader XBB wave [63].

The dynamic mutational landscape presents ongoing challenges and uncertainties. Essabbar et al. [65] report that future dominant strains are likely to emerge from recombinant strains, emphasizing the need for continued genomic surveillance, global data sharing, and collaborative research efforts. The concern with mutational dynamics also applies to understanding the evolution of mutations in the virus. In our results, the prevalence of XBB sublineages is noteworthy. At the local and national levels, dissemination of XBB in Brazil during 2023 was driven by stepwise spike mutations that increased ACE2 affinity and immune escape, which underscores the need to maintain real time genomic surveillance capacity [66]. At the global level, risk assessments reported increased transmissibility and immune escape for XBB.1.5, and public health agencies updated vaccine composition to XBB.1.5 based formulations, with early estimates indicating protection of the updated vaccine against symptomatic infection in adults [67, 68].

In conclusion, our study sheds light on the molecular epidemiology of SARS-CoV-2 in Tocantins State, Brazil, from December 2021 to June 2023, highlighting the predominant presence of the XBB.1.18.1 lineage. Despite its emergence from international sources, our findings suggest that Tocantins played a pivotal role in the dissemination of this lineage within Brazil, consistent with extensive trade routes and high human mobility along the trans-Brazilian highway BR-153. However, the study has limitations. While our analysis provides valuable insights into the local and international spread of the XBB.1.18.1 lineage, the study’s scope is limited to genomic and epidemiological data, and further investigations are warranted to elucidate additional factors influencing virus transmission dynamics. We also acknowledge potential temporal bias due to uneven sequencing over time; to mitigate this, we fixed the analysis window a priori and removed temporal outliers after root-to-tip screening, interpreting node ages and diffusion rates with appropriate caution. Additionally, the rapid evolution of SARS-CoV-2 variants and the emergence of new sublineages pose ongoing challenges, underscoring the importance of continuous genomic surveillance to inform public health interventions and control measures effectively.

## Supplementary Information

Below is the link to the electronic supplementary material.

Supplementary Material 1 (DOCX 993 KB)

Supplementary Material 2 (PDF 19.4 KB)

Supplementary Material 3 (PDF 19.3 KB)

## Data Availability

Genome data were obtained from GISAID under its data access agreement (sequences available up to 30 September 2023). All newly generated Tocantins genomes have been submitted to GISAID; accession identifiers are listed in Supplementary Table S1 (GISAID Identifier: EPI_SET_251003so). To ensure reproducibility, we also provide a GISAID EPI_SET that bundles the final dataset of 1,073 sequences used in the phylogenetic analysis (GISAID Identifier: EPI_SET_251003pd; see Supplementary Table S2). The repository with the study’s machine-readable metadata, BEAST XML files, and BEAST log files is available at: (https://github.com/Ueric/A-Comprehensive-Analysis-of-the-SARS-CoV-2-Omicron-Variant-in-Tocantins-State-Brazil.git). No raw sequence data are redistributed, in accordance with GISAID’s terms of use.
