# Supplementary material for: A comprehensive analysis of the SARS-CoV-2 omicron variant in Tocantins State, Brazil, and tracing the spread of the XBB.1.18.1 lineage
Source: Braz J Microbiol. 2026 Mar 23;57(1):89. doi: 10.1007/s42770-026-01884-1 (PMC13009458; doi:10.1007/s42770-026-01884-1)
Supplement: Supplementary file 1 — Supplementary Material 1 (DOCX 993 KB) [file 42770_2026_1884_MOESM1_ESM.docx]

**A Comprehensive Analysis of the SARS-CoV-2 Omicron Variant in Tocantins State, Brazil, and Tracing the Spread of the XBB.1.18.1 Lineage**

Mateus Silva Santos^1,2#^, Ueric José Borges de Souza^1#*^, Franciano Dias Pereira Cardoso^3^, Jucimária Dantas Galvão^3^, Fernando Rosado Spilki^4^, Célia Maria de Almeida Soares^2,5^, Fabrício Souza Campos^1,6*^

1 - Bioinformatics and Biotechnology Laboratory, Campus of Gurupi, Federal University of Tocantins, Gurupi, Tocantins, 77410-570, Brazil

2 - Tropical Medicine Post-Graduate Program – Federal University of Goiás, Goiânia, Goiás, 74690-900 Brazil.

3 - Central Public Health Laboratory of the State of Tocantins, Palmas, 77054-970, Brazil

4 - Molecular Microbiology Laboratory, Feevale University, Novo Hamburgo 93525-075, Brazil

5 - Molecular Biology Laboratory, Institute of Biological Sciences, Federal University of Goiás, Goiânia, Goiás, 74690-900 Brazil.

6 - Virology Laboratory, Department of Microbiology, Immunology, and Parasitology, Institute of Basic Health Sciences, Federal University of Rio Grande do Sul, Porto Alegre 90050-170, Brazil

#Both authors contributed equally.

*Corresponding author: [uericjose@gmail.com](mailto:uericjose@gmail.com) (U.J.B.S.), [camposvet@gmail.com](mailto:camposvet@gmail.com) (F.S.C.).

**SUPPLEMENTARY MATERIAL**


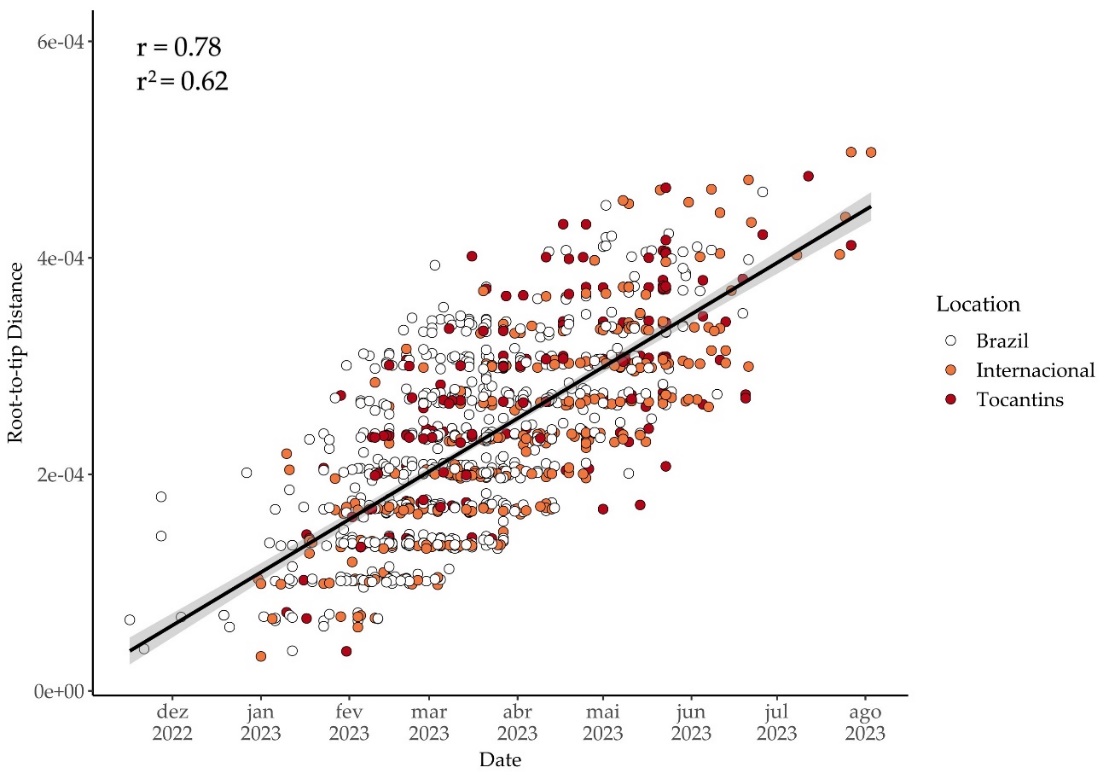


**Supplementary Figure 1.** Root-to-tip regression of genetic distances and sampling dates for 1073 SARS-CoV-2 Genomes of the XBB.1.18.1 Lineage Across Global Regions. Correlation coefficient (r) and r squared are depicted above the graph.


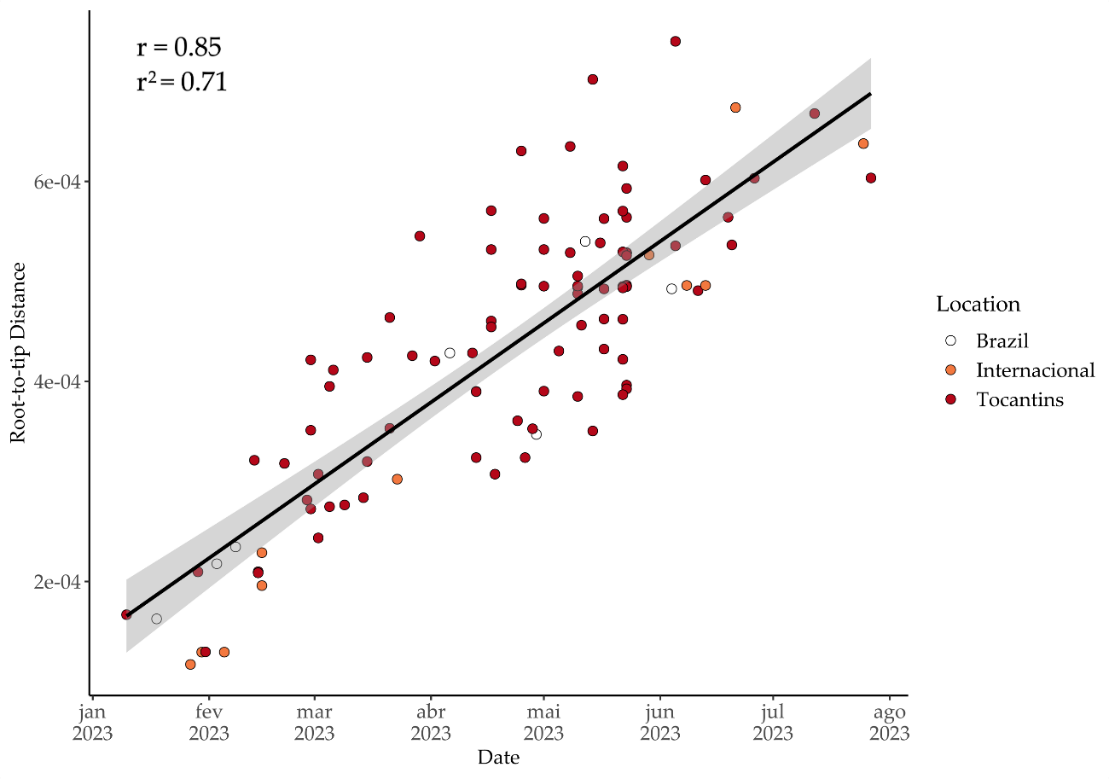


**Supplementary Figure 2**. Root-to-tip regression of genetic distances and sampling dates for 106 XBB.1.18.1 SARS-CoV-2 Clade I Samples Across Global Regions. Correlation coefficient (r) and r squared are depicted above the graph.


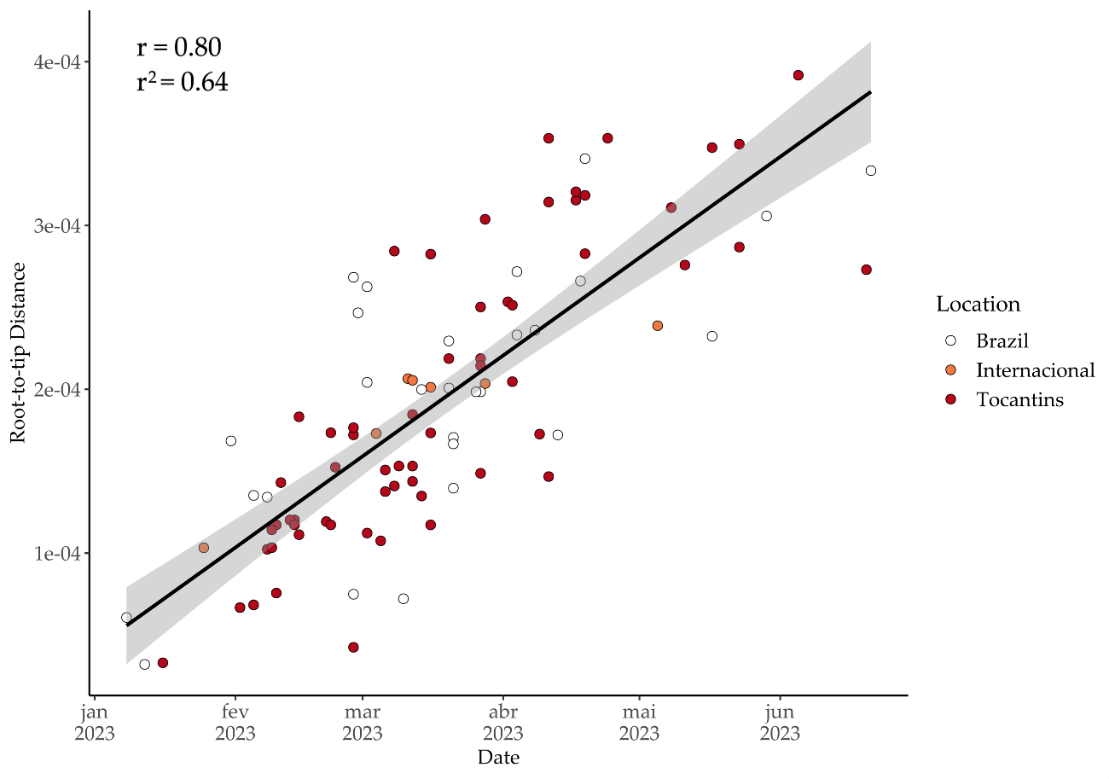


**Supplementary Figure 3**. Root-to-tip regression of genetic distances and sampling dates for 96 XBB.1.18.1 SARS-CoV-2 Clade II Samples Across Global Regions. Correlation coefficient (r) and r squared are depicted above the graph.


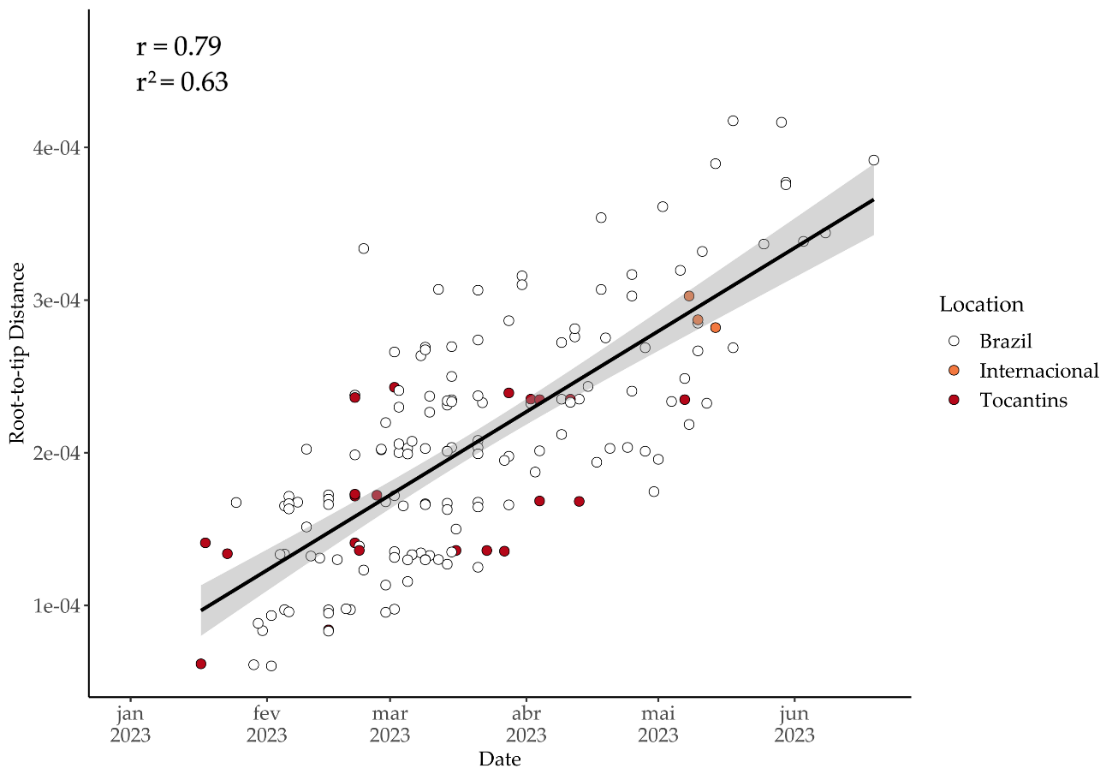


**Supplementary Figure 4.** Root-to-tip regression of genetic distances and sampling dates for 132 XBB.1.18.1 SARS-CoV-2 Clade III Samples Across Global Regions. Correlation coefficient (r) and r squared are depicted above the graph.


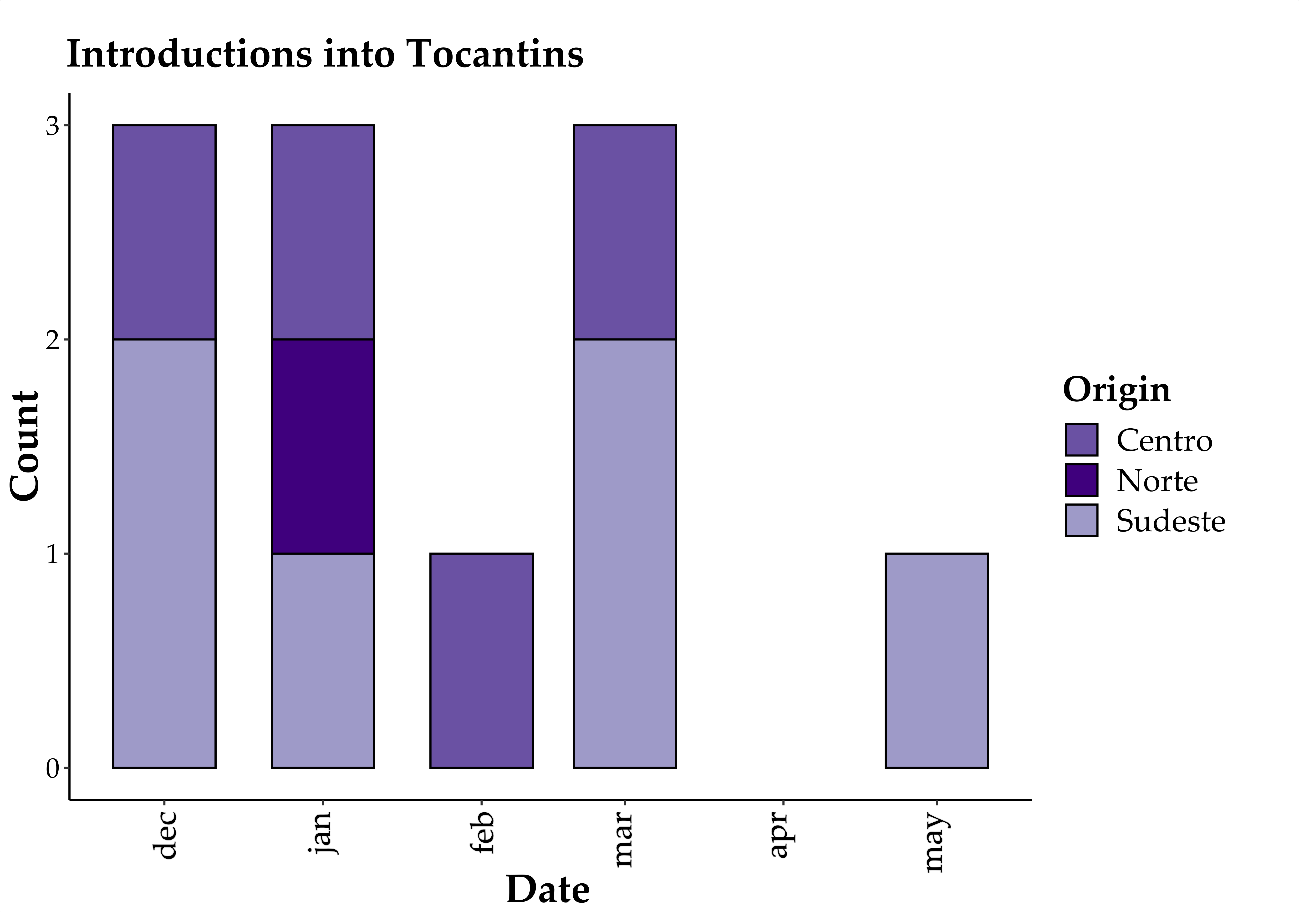


**Supplementary Figure 5**. Temporal Distribution of Regions Importing the XBB.1.18.1 SARS-CoV-2 Lineage in Tocantins State. Legends in the graphs indicate the corresponding regions of origin.


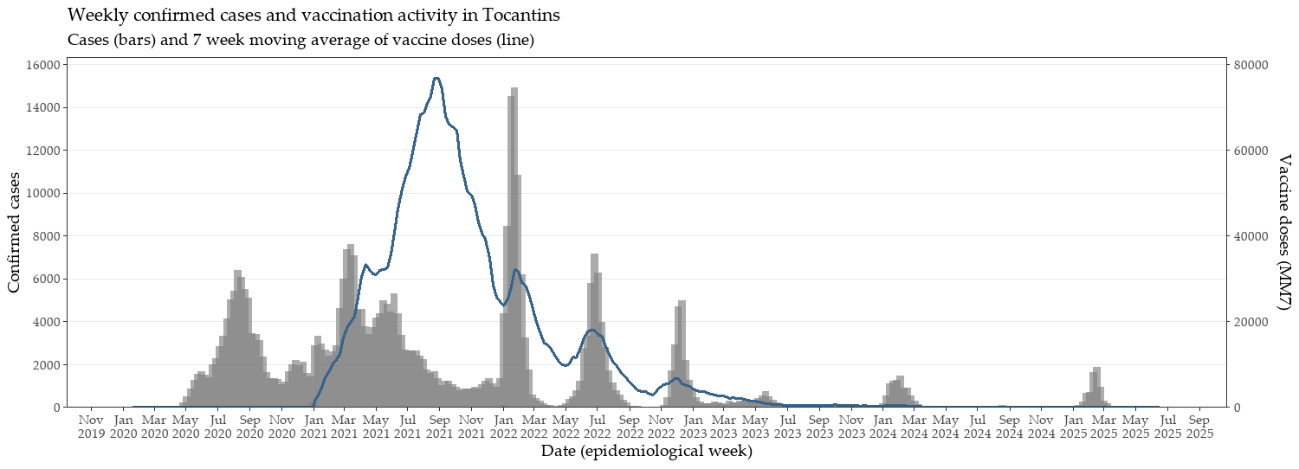


**Supplementary Figure 6**. Weekly confirmed cases and vaccination activity in Tocantins. Bars show weekly confirmed COVID 19 cases; the line represents the 7-week moving average of vaccine doses administered (right axis). The timeline highlights the rapid vaccination scale up in 2021 and subsequent heterogeneous activity through 2022–2024, alongside successive epidemic waves. Data derived from the official state surveillance platform Integra Saúde Tocantins (<https://integra.saude.to.gov.br/covid19>).

**Supplementary Table S1**. Newly generated SARS-CoV-2 genomes from Tocantins and their corresponding GISAID accession identifiers.

Available on: https://github.com/Ueric/A-Comprehensive-Analysis-of-the-SARS-CoV-2-Omicron-Variant-in-Tocantins-State-Brazil.git

**Supplementary Table S2**. Complete dataset of 1,073 SARS-CoV-2 sequences used in the phylogenetic analysis (GISAID EPI_SET_251003pd).

Available on: https://github.com/Ueric/A-Comprehensive-Analysis-of-the-SARS-CoV-2-Omicron-Variant-in-Tocantins-State-Brazil.git
