# Supplementary material for: A comprehensive analysis of the SARS-CoV-2 omicron variant in Tocantins State, Brazil, and tracing the spread of the XBB.1.18.1 lineage
Source: Braz J Microbiol. 2026 Mar 23;57(1):89. doi: 10.1007/s42770-026-01884-1 (PMC13009458; doi:10.1007/s42770-026-01884-1)
Supplement: Supplementary file 3 — Supplementary Material 3 (PDF 19.3 KB) [file 42770_2026_1884_MOESM3_ESM.pdf]

SUPPLEMENTAL TABLE

Data Availability

GISAID Identifier:	EPI_SET_251003pd
DOI:	https://doi.org/10.55876/gis8.251003pd

All genome sequences and associated metadata in this dataset are published in GISAID's EpiCoV database. To view the contributors of each individual sequence with details such as accession number, Virus name, Collection date, Originating Lab and Submitting Lab and the list of Authors, visit EPI_SET_251003pd

Data Snapshot

EPI_SET_251003pd is composed of 1,073 individual genome sequences.
The collection dates range from 2022-11-17 to 2023-08-03;
Data were collected in 37 countries and territories.
